# Magnolin Mitigates Skin Ageing Through the CXCL10/p38 Signalling Pathway

**DOI:** 10.1111/jcmm.70507

**Published:** 2025-05-19

**Authors:** Cheng Wang, Xiaoyun Hu, Tianlin Song, Fan Hu, Le Du, Chenqiong Yan, Tianwei Shen, Nihong Li, Wei Yang, Li Li, Nian Deng, Xingwu Jiang, Yelin Wu, Rui Ye

**Affiliations:** ^1^ Shanghai Tenth People's Hospital, Tongji University School of Medicine Shanghai China; ^2^ UNISKIN Research Institute on Skin Aging, Inertia Shanghai Biotechnology Co., Ltd. Shanghai China; ^3^ DermaHealth Shanghai Biotechnology Co., Ltd. Shanghai China; ^4^ Department of Dermatology & Venerology, Center of Cosmetic Safety and Efficacy Evaluation and NMPA Key Laboratory for Human Evaluation and Big Data of Cosmetics West China Hospital, Sichuan University Chengdu China; ^5^ Department of Materials Science and State Key Laboratory of Molecular Engineering of Polymers Fudan University Shanghai China

**Keywords:** cell senescence, chemokine, CXCL10, magnolin, skin ageing

## Abstract

Skin ageing accelerates systemic ageing and impairs the body's disease resistance. Inflammaging is a significant contributor to skin ageing. However, the mechanism by which inflammatory factors contribute to skin ageing remains unclear. We conducted screenings and identified CXC motif chemokine ligand 10 (CXCL10) as an inducer of skin ageing, which can be effectively inhibited by magnolin extracted from *Magnolia biondii* flower. Our results show that CXCL10 not only enhances beta‐galactosidase (β‐gal) activity but also upregulates the expression of p21/p16, along with matrix metalloproteinases (MMP1, MMP9 and MMP10), through activation of the C‐X‐C motif chemokine receptor 3 (CXCR3) in human primary fibroblasts. These findings suggest that CXCL10 drives skin ageing by inducing cellular senescence and extracellular matrix degradation. Importantly, treatment with magnolin significantly mitigates skin ageing phenotypes induced by CXCL10 via the p38 signalling pathway. Furthermore, our study demonstrates that magnolin significantly mitigates UV‐induced skin ageing in ex vivo human skin samples as well as on human facial skin. This study provides insight into the role of chemokine CXCL10 in promoting inflammaging and proposes an innovative approach for preventing and treating skin ageing.

## 1 Introduction

Skin ageing is a complex process characterised by the degradation of skin tissue structure and function resulting from prolonged exposure to both internal and external stressors [1]. Throughout this ageing process, various alterations in skin texture can be observed, including decreased elasticity, thickness, tension and changes in surface texture. Moreover, pigmentation changes, as well as vascular atrophy or hyperplasia, may also manifest [2, 3, 4]. The impact of skin ageing extends beyond cosmetic concerns; it poses significant medical challenges such as compromised protection against pathogens, delayed wound healing and an increased risk of cancer [5]. Furthermore, skin ageing can accelerate systemic ageing processes, leading to physiological and pathological changes throughout the body [6, 7]. Therefore, it is crucial to investigate the underlying mechanisms of skin ageing and develop effective strategies for its prevention and treatment.

Chronic, low‐grade inflammation is a well‐established contributor to skin ageing [5, 8]. The skin serves as the primary barrier protecting our body against external harmful substances. Exogenous factors such as sunlight exposure, environmental pollution, excessive use of cosmetics and psychological stress can induce chronic inflammation in the skin [9, 10]. This persistent inflammatory state can clinically manifest as sensitive skin, acne vulgaris, contact dermatitis and other conditions [11, 12]. Oxidative damage [13], DNA damage and telomere dysfunction resulting from chronic inflammation can expedite the process of skin ageing [14]. Furthermore, senescent aged skin cells secrete senescence‐associated secretory phenotype (SASP) factors that perpetuate a detrimental cycle of inflammation and ageing [1, 15]. Therefore, targeting chronic inflammation may serve as a preventive measure against multifactorial‐induced skin ageing. However, the specific inflammatory factors that significantly contribute to skin ageing remain unclear.

Chemokines are a class of inflammatory factors that mediate targeted chemotaxis of immune cells to eliminate harmful substances, thereby maintaining protective immunity. However, excessive recruitment of inflammatory cells can lead to chronic inflammation, which contributes to age‐related diseases. Additionally, ageing cells express and secrete various chemokines [1, 16]. These findings suggest the potential crucial role of chemokines in inflammation‐induced skin ageing. Recent research has demonstrated that the chemokine CXCL9 can induce cellular senescence in vascular endothelial cells [17], indicating a potential involvement of chemokines in cardiovascular ageing. Nevertheless, limited studies have investigated the functions of chemokines in skin ageing.

In this study, we conducted a screening of various inflammatory factors to determine their potential in inducing skin ageing. Our findings revealed that the chemokine CXCL10 significantly induces skin fibroblast senescence and matrix metalloproteinase (MMPs) expression. Subsequently, we identified magnolin, a natural product derived from *Magnolia biondii* flower extract, as an effective inhibitor of CXCL10‐induced skin ageing both ex vivo on human skin samples and in vivo on human facial skin. These results provide novel strategies for the prevention and treatment of skin ageing.

## 2 Materials and Methods

### 2.1 Cell Culture

Human skin fibroblasts (HSFs) were obtained from Guangdong Biocell Biotechnology Co. Ltd. HSFs were derived from the abdominal skin of a female donor. The isolation and culture process were as follows: The processed tissue was placed in DMEM medium containing collagenase and incubated at 4°C overnight. Subsequently, an equal volume of trypsin digestion solution was added, and the digestion was terminated after 20 min at 37°C. The cells were collected by centrifugation at 1000 rpm for 10 min. After adjusting the cell density with fibroblast culture medium, the cells were seeded into T75 flasks and cultured in DMEM supplemented with 10% FBS and 1% penicillin–streptomycin solution at 37°C in a humidified atmosphere of 95% air and 5% CO_2_.

### 2.2 Reagents and Antibodies

IL‐1α, IL‐1β, IL‐4, IL‐10, IL‐12, IL‐13, IL‐23, IL‐27 and IL‐33 were purchased from PeproTech (New Jersey, USA). IL‐8, IL‐18, IL‐22, CXCL1, CXCL10, CCL18 and IFNγ were obtained from SinoBiological (Beijing, China). Anti‐p16 (ab51243), anti‐p21 (ab109520) and anti‐Lamin B1 (ab8982) were purchased from Abcam (Cambridge, MA, USA). Anti‐GAPDH (ab0036), anti‐P‐p38 (cy6391) and anti‐p38 (cy5488) were purchased from Abways (Shanghai, China). *Phaeodactylum trigonalis* and hydrolysed rhodophycea extract were purchased from Greenaltech (Spain). *Magnolia biondii* flower extract, *Aesculus chinensis* extract and *bifida ferment* lysate were purchased from JAKA (Shanghai, China). *Adenium obesum* and *Eryngium maritimun* extracts were purchased from Acelbio (Tianjin China). *Tephrosia purpurea* , *Glucosylrutin*, *Rutin* and Diglucosyl gallic acid were purchased from Givaudan (Switzerland). Glycerophosphoinositol choline was purchased from IRB (China). *Ophiopogon japonicus root* extract, *Mentha piperita leaf* extract and *Salvia miltiorrhiza* root extract were purchased from Silab (France). *Centaurea Cyanus flower* extract was purchased from Merck (USA). *Aframomum melegueta* extract was purchased from IDBIO SAS (France). *Sphingomonas ferment* extract was purchased from CLR (Berlin, Germany). Isoquercitrin, Quercetin phospholipid and Quercetin were purchased from Sigma (USA).

### 2.3 Measurement of Cell Senescence

HSF cells were seeded at 1 × 10^5^ cells/mL in a 6‐well plate with 2 mL in each well and cultured at 37°C for 24 h. After that, the cells were treated with designed compounds for another 24 h. Then the cells were collected, lysed and sonicated using a lysis buffer. The activity of β‐gal was measured using the β‐gal Activity Assay Kit (Solarbio, Beijing, China), while the cells were also stained using the Senescence β‐gal Staining Kit (Beyotime, Shanghai, China) and visually observed by taking photographs.

### 2.4 Real‐Time Quantitative Reverse‐Transcriptase‐PCR

HSF cells were seeded at a concentration of 1 × 10^5^ cells/mL in a 6‐well plate with 2 mL of culture medium and incubated at 37°C for 24 h. The cells were then treated with different compounds for another 24 h. After treatment, the cells were washed with PBS once, and total RNA was extracted by lysing the cells with Trizol. Next, the RNA was transcribed into cDNA using a reverse transcriptase kit (Takara, Japan). Real‐time quantitative reverse‐transcriptase PCR analysis was performed using specific primers (Table S1) and SYBR Green real‐time PCR reagents (Takara, Japan), following the manufacturer's instructions.

### 2.5 Identification and Purification of Magnolin

To further investigate the composition of *Magnolia biondii* flower extract, HPLC analysis was performed, and a peak of high abundance at a retention time of 6.021 min was observed. Subsequently, in order to confirm this substance, HPLC analysis on a standard sample of magnolin was carried out under the same HPLC conditions. The HPLC peak at the same retention time was observed, confirming that the substance was indeed magnolin. Finally, we separated and purified this substance by the preparative HPLC method, obtaining the desired magnolin. High‐resolution mass spectrometry (HR‐MS) was carried out, and the obtained result was as follows: HR‐MS (ESI, m/z): calcd for C_23_H_29_O_7_ [M + H]^+^, 417.1913, found 417.19113. Moreover, ^1^H NMR spectroscopy was also performed, and the NMR data were found to be in accordance with those of the target compound Magnolin.

### 2.6 Cell Cycle Assays

HSF cells were seeded at 1 × 10^5^ cells/mL in a 6‐well plate with 2 mL in each well and cultured at 37°C for 24 h. Afterward, cells were treated with the desired compound for 24 h, they were harvested by trypsinization, fixed in ice‐cold 70% ethanol, and stored at 4°C for 1 h. Next, cells were centrifuged at 300*g* for 5 min and washed with PBS. Then cells were resuspended in 500 μL of staining buffer containing 0.5% Tween 20, 50 μg/mL RNase and 20 μg/mL propidium iodide (PI, Sigma, Missouri, USA). Then the cell suspension was passed through a 40‐μm cell strainer to remove clumps and was subjected to flow cytometry analysis. The DNA content of the cells was measured by propidium iodide staining, which allows for the identification of the different phases of the cell cycle (G_0_/G_1_, S and G_2_/M). The percentage of cells in each phase of the cell cycle was calculated.

### 2.7 Measurement of Cell Viability

To measure cell viability, HSF cells were seeded in a 96‐well plate with a density of 4 × 10^4^ cells/mL and cultured at 37°C for 24 h. After that, different treatments were added to the cells for an additional 24 h. Then, 10 μL of CCK‐8 solution (Beyotime, Shanghai, China) was added to each well and incubated for 2 h at 37°C. The absorbance of each well was measured at 450 nm using a microplate reader. The level of cell viability was determined by calculating the absorbance of each well and comparing it with the control group.

### 2.8 Western Blot Analysis

After the HSF cells were treated with the desired compound for 24 h, the cells were lysed with RIPA lysis buffer (P0013B) (Beyotime, Shanghai, China) to collect the total protein. The protein concentration was measured using a BCA protein concentration assay kit (P0010) (Beyotime, Shanghai, China). An equal amount of protein was separated using SDS‐PAGE, transferred to NC membranes, blocked with 5% skim milk powder for 1 h, incubated with a specific primary antibody at 4°C overnight and then incubated with an HRP‐labelled secondary antibody for 2 h. A Western blot detection system (Tanon‐5200) was used for imaging.

### 2.9 Immunofluorescence Staining

To perform immunofluorescence staining, HSF cells were seeded at a density of 4 × 10^4^ cells/dish in 15 mm glass‐bottomed culture dishes. After 24 h of culture, the cells were treated with different compounds for 24 h. Afterward, the cells were fixed with 4% paraformaldehyde and incubated with 5% BSA at room temperature for 30 min. The cells were then incubated with primary antibodies, rabbit anti‐p21, mouse anti‐Lamin B1, rabbit anti‐p16, and mouse anti‐Lamin B1 antibodies, overnight at 4°C, respectively. Then, the cells were incubated with anti‐rabbit Cy3‐conjugated and anti‐mouse FITC‐conjugated secondary antibodies at room temperature for 2 h. Finally, DAPI (5 μg/mL) was added to the cells and incubated at room temperature for 5 min. The stained cells were observed and imaged using a confocal microscope (Zeiss, Oberkochen, Germany).

### 2.10 Enzyme‐Linked Immunosorbent Assay

HSF cells were seeded at 1 × 10^5^ cells/mL in a 6‐well plate with 2 mL in each well and cultured at 37°C for 24 h. Then, different treatments were added to the cells for 24 h. After treatment, the supernatant was collected for analysis of the cytokines IL‐1β, CCL5, PAI‐1, MCP‐1, IL‐8, HGF, MMP9 and MMP10. The levels of these cytokines were measured using commercial ELISA kits (Thermo, USA) according to the manufacturer's instructions.

### 2.11 Ex Vivo Skin Culture

The ex vivo human skin was donated by volunteers who had signed an informed consent. Access to biopsy material was in accordance with Chinese law and satisfied the requirements of the local Ethics Committee. The freshly obtained skin tissue was immersed in 75% alcohol, subjected to a 30 s wash, followed by three washes with sterile PBS buffer. Subsequently, the skin was cut into pieces measuring approximately 24 ± 2 mm^2^ and cultured in a 5% CO_2_ incubator at 37°C. After 1 day of culture, the isolated skin tissue was exposed to UVA (30 J/cm^2^) and UVB (50 mJ/cm^2^) radiation for three consecutive days, either with or without administration. Finally, the skin tissue was collected and fixed using 4% paraformaldehyde for H&E staining, Victoria Blue staining, Masson staining and immunofluorescence staining.

### 2.12 Clinical Evaluation

The clinical research was conducted at ICAS Testing Technology Service (Shanghai) Co. Ltd. The research protocol was reviewed and approved by the ICAS Ethics Committee for Clinical Research under project number E2023158. Prior to participation, all potential benefits, risks and possible complications were thoroughly explained to the subjects. Informed consent was obtained from all participants, who voluntarily agreed to take part in the study. The inclusion criteria were as follows: (1) healthy women, aged 18–40 years old, with sensitive skin (lactic acid stinging score ≥ 3), facial redness, dry itching and stinging, skin laxity, eye wrinkles ≥ 2, weak skin barrier (TWEL ≥ 15 g/h/m^2^); (2) capable of maintaining a regular lifestyle during the study; (3) agreed not to use any cosmetics, drugs and health products that may affect the results during the trial.

Basic skin values of subjects were assessed at 0, 7, 14, 28 and 56 days using VisioScan VC20 plus (CK, Germany) for capturing photos and analysing SEr value for skin roughness parameter. VISIA images (Canfield USA) were utilised to collect and analyse the proportion of wrinkles under the eyes. Cutometer dual MPA580 (CK Germany) was employed to measure skin elasticity (R2), skin firmness (F4) and skin distensibility (R0). Skin glossiness was measured using a Glossymeter, while epidermal density was determined by the Ultrascan UC22 device from CK Germany.

### 2.13 Statistical Analysis

Data were presented as mean ± SEM in this study. Statistical analysis was conducted using GraphPad Prism 8 (GraphPad Software Inc., San Diego, California, USA). Multiple‐group comparisons were performed using one‐way ANOVA followed by Tukey's post hoc tests. For clinical evaluation data, which comprise paired samples, the analysis proceeds as follows: a paired *T*‐test was conducted if the samples exhibit a normal distribution. In contrast, if the samples deviate from a normal distribution, the Wilcoxon signed‐rank test was utilised. *P* values < 0.05 were considered statistically significant, and ‘ns’ indicated no significant difference.

## 3 Results

### 3.1 CXCL10 Induces Skin Fibroblast Senescence

**FIGURE 1:**
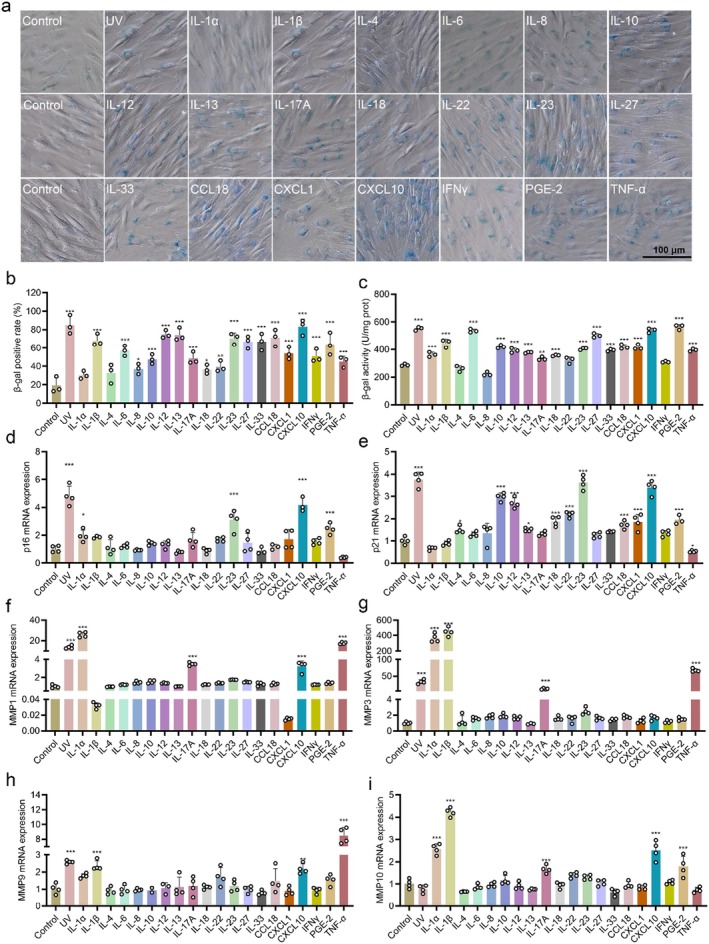
Inflammatory cytokines induce skin fibroblast senescence and the expression of MMPs in HSF cells. (a) β‐gal staining and imaging. (b) Quantification of β‐gal staining. (c) Detection of β‐gal activity. QPCR detection of (d) p16, (e) p21, (f) MMP1, (g) MMP3, (h) MMP9 and (i) MMP10 mRNA expression. HSF cells were treated with 100 ng/mL of inflammatory cytokines for 24 h and the positive control was exposed to 60 mJ/cm^2^ of UVB radiation. (*n* = 3, the treatment groups were compared with the control group. *p < 0.05, **p < 0.01, ***p < 0.001).

Continuous stimulation of the skin by physical, chemical, psychological, mechanical and microbial factors leads to the secretion of various inflammatory factors, resulting in chronic inflammation and skin ageing known as inflammaging. However, it remains unclear which specific inflammatory factors directly contribute to ageing. Based on previous research reports, we selected 20 inflammatory factors that were significantly upregulated in skin cells following exposure to stimuli such as UV radiation, microbial infection, stress and air pollution [18, 19, 20, 21, 22, 23, 24, 25, 26]. We investigated their impact on human skin fibroblasts (HSFs) ageing. β‐galactosidase (β‐gal) staining experiments showed that most inflammatory factors can induce an increase in β‐gal expression (Figure 1a). Quantitative analysis of β‐gal staining demonstrated that IL‐1β, IL‐12, IL‐13, IL‐23, IL‐27, IL‐33, CCL18, CXCL10 and PGE‐2 can elevate β‐gal levels by approximately threefold in fibroblasts (Figure 1b). Meanwhile, CXCL10, PGE‐2 and IL‐6 can enhance the activity of β‐gal by approximately twofold (Figure 1c). Moreover, we conducted an additional investigation into the expression of two other genes associated with skin ageing, namely p21 and p16 [27, 28]. Our findings revealed that IL‐1α, IL‐1β, IL‐23, CXCL10 and PGE‐2 display a significant capacity to stimulate the transcription of p16. Similarly, we observed that IL‐10, IL‐12, IL‐23 and CXCL10 exhibit a substantial ability to induce the transcription of p21 (Figure 1d,e). Furthermore, we also examined the effects of these inflammatory factors on the cell cycle and observed that IL‐17A, IL‐33, CXCL10 and PGE‐2 have an ability to induce cell cycle arrest at the G_1_ transition (Figure S1). These findings suggest a crucial role for CXCL10 in inducing fibroblast senescence, a key factor contributing to inflammaging.

In addition to displaying conventional cellular senescence features, skin ageing is characterised by a specific reduction in extracellular matrix components such as collagen and elastin. Matrix metalloproteinases (MMPs) play a crucial role in the degradation of collagen and elastin. Therefore, we investigated the impact of inflammatory factors on MMP1, MMP3, MMP9 and MMP10 expression. Our findings demonstrated that IL‐1α, IL‐17A, CXCL10 and TNF‐α significantly induced the transcription of MMP1; IL‐1α, IL‐1β, IL‐17A and TNF‐α induced the transcription of MMP3; IL‐1β, CXCL10 and TNF‐α induced the transcription of MMP9; while IL‐1α, IL‐1β, IL‐17A, CXCL10 and PGE‐2 stimulated the transcriptional activity of MMP10 (Figure 1f–i). In summary, in addition to well‐known inflammatory factors like IL‐1α, IL‐1β and TNF‐α, CXCL10 can also significantly stimulate the expression of MMPs.

### 3.2 CXCL10 Induces HSF Cell Senescence and the Expression of MMPs Through CXCR3

**FIGURE 2:**
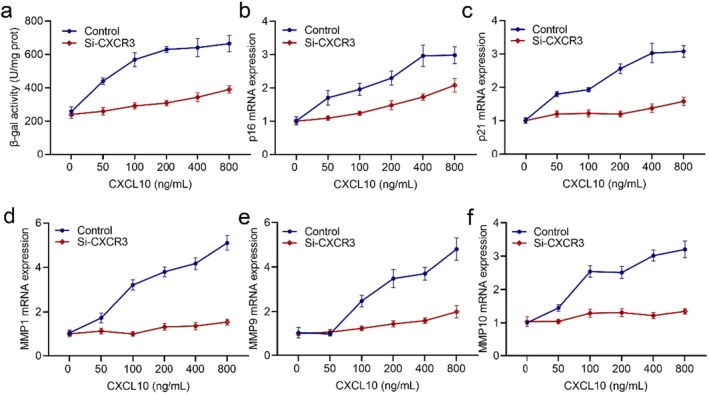
CXCL10 induces HSF cell senescence and the expression of MMPs through CXCR3. (a) β‐gal activity assay. QPCR detection of (b) p16, (c) p21, (d) MMP1, (e) MMP9 and (f) MMP10 mRNA expression. HSF cells treated with different concentrations of CXCL10 in the presence or absence of siCXCR3 for 24 h, and total RNA was extracted for reverse transcription (*n* = 3).

To further validate the role of CXCL10 in promoting skin ageing in HSF cells, we conducted an experiment in which the cells were exposed to different concentrations of CXCL10. Concurrently, we employed siRNA to specifically silence CXCR3, the receptor for CXCL10. As depicted in Figure 2, a dose‐dependent upregulation of p16, p21, MMP1, MMP9 and MMP10 gene expression was observed upon treatment with CXCL10. However, this induction was attenuated following the administration of CXCR3 siRNA. These findings collectively provide compelling evidence that CXCL10 induces senescence and the expression of MMPs in HSF cells through activation of the CXCR3.

### 3.3 Magnolin Suppresses p38 Phosphorylation to Inhibit CXCL10‐Induced Cellular Senescence

**FIGURE 3:**
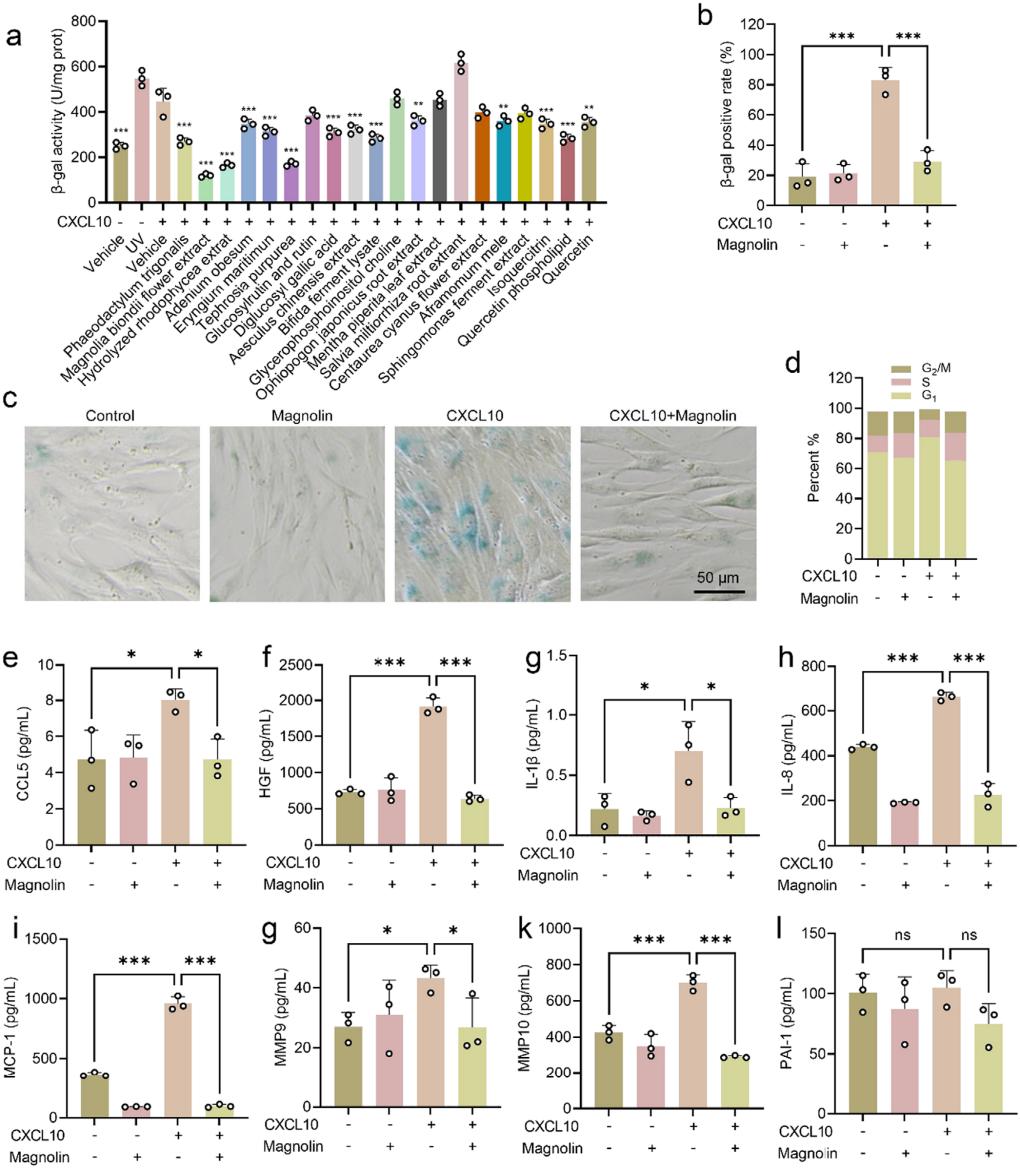
Magnolin inhibits CXCL10‐induced cell senescence phenotypes. (a) Screening of potential plant extracts that effectively inhibit CXCL10‐induced β‐gal activity. (Comparison of the CXCL10 group against the other groups.) (b, c) Quantification of β‐gal staining and the representative images. (d) The cell cycle was analysed by propidium iodide (PI) staining and flow cytometry. (e–l) HSF cells were seeded in culture dishes for 24 h and then treated with 5 μg/mL magnolin for 24 h in the presence of 200 ng/mL CXCL10 to induce cellular senescence. The levels of SASP factors released into the culture medium were measured by ELISA. (*n* = 3, *p < 0.05, **p < 0.01, ***p < 0.001).

Since CXCL10 has been identified as a pivotal factor contributing to skin inflammaging, we screened various plant extracts with potential anti‐inflammatory properties and evaluated their ability to inhibit CXCL10‐induced cellular senescence and MMPs expression at safe concentrations (Figure S2). Our findings revealed that *Magnolia biondii* flower extract, *hydrolyzed rhodophycea* extract and *Tephrosia purpurea* significantly attenuated β‐gal activity (Figure 3a). Considering the superior efficacy of *Magnolia biondii* flower extract, HPLC was performed to isolate and purify the extract and magnolin finally was obtained. In order to validate the substance as Magnolin, high‐resolution mass spectrometry (HR‐MS) was performed, and a molecular ion peak with an m/z value of 417.19113 was detected, which is consistent with the calculated molecular weight of C_23_H_29_O_7_ [M + H]^+^ (417.1913), thereby confirming the presence of the target compound (Figure S3). Moreover, ^1^H NMR spectroscopy was also performed, and the ^1^H NMR data were found to be in accordance with those of the target compound Magnolin (Figure S3). The results revealed magnolin as the predominant compound in *Magnolia biondii* flower extract, suggesting its potential as a key anti‐ageing constituent (Figure S3). Next, we cultured HSF cells with magnolin for 24 h and found that the safe concentration is 5 μg/mL (Figure S4). We selected this concentration for subsequent experiments.

To further investigate the function of magnolin in CXCL10‐induced skin inflammaging, we treated the HSF cells with different concentrations of CXCL10 in the presence or absence of magnolin. As shown in Figure 3b,c, treatment with magnolin significantly reduced the percentage of CXCL10‐induced β‐gal positive cells by 64.7%. Moreover, we observed that magnolin treatment can effectively reverse the G_1_ arrest induced by CXCL10 in HSF cells (Figure 3d and Figure S5). Another characteristic feature of senescent cells is an elevation in SASP secretion. The results showed that magnolin treatment significantly reduced SASP secretion, including CCL5, HGF, IL‐1β, IL‐8, MCP‐1, MMP9 and MMP10 (Figure 3e–l), further demonstrating that magnolin from the *Magnolia biondii* flower extract can inhibit CXCL10‐induced cellular senescence and MMP expression.

**FIGURE 4:**
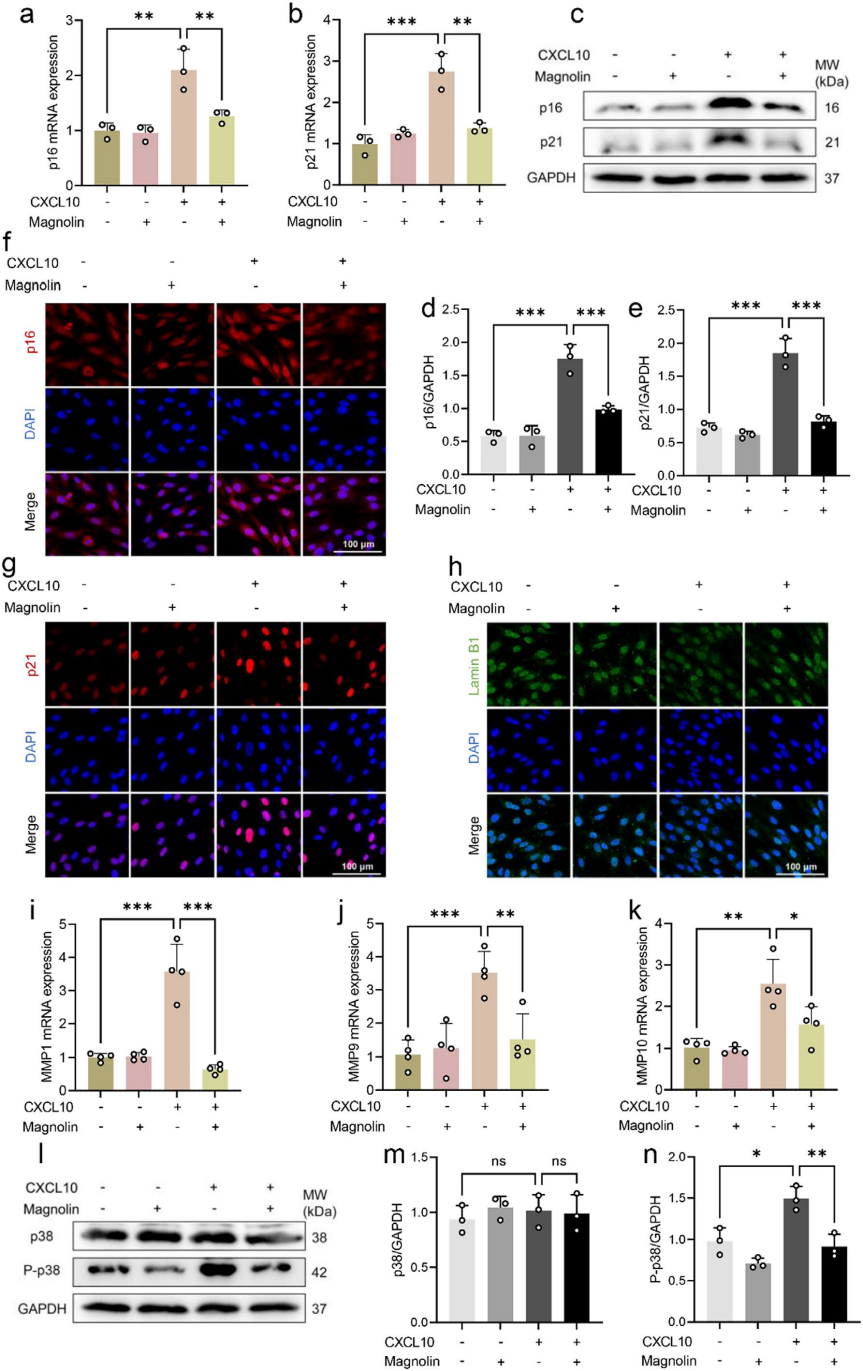
Magnolin inhibits CXCL10‐induced cellular senescence and MMPs expression by suppressing p38 phosphorylation. (a, b) QPCR detection of p16 and p21 mRNA expression. (c) Western blot detection of the p16 and p21 protein expression, GAPDH was used as an internal control. (d, e) Quantification of the results from Figure 4c. (f–h) Immunofluorescence staining of p16, p21 and Lamin B1. (i‐k) QPCR detection of MMP1, MMP9 and MMP10 mRNA expression. (l) Western blot detection of the expression of p38 and P‐p38. (m–n) Quantification of the results from Figure 4l. HSF cells were seeded in culture dishes for 24 h and then treated with 5 μg/mL magnolin for 24 h in the presence of 200 ng/mL CXCL10 to induce cellular senescence. (*n* = 3, *p < 0.05, **p < 0.01, ***p < 0.001).

To further investigate the inhibitory mechanism of magnolin on cellular senescence, we examined the alterations in key ageing‐related proteins and observed that magnolin effectively suppressed the expression of p16 and p21 at both mRNA and protein levels (Figure 4a–e). Consistently, immunofluorescence staining revealed that magnolin significantly reduced the expression of p16 and p21 while increasing Lamin B1 expression. These findings suggest that magnolin can inhibit CXCL10‐induced upregulation of p16 and p21, as well as prevent CXCL10‐mediated downregulation of Lamin B1, thereby decelerating cellular senescence (Figure 4f–h). Furthermore, magnolin also exhibited inhibitory effects on CXCL10‐induced MMP expression, indicating its ability to not only impede CXCL10‐driven cellular senescence but also prevent ECM degradation (Figure 4i–k). Previous studies have demonstrated that phosphorylation of p38 plays a crucial role in inducing cellular senescence and MMP expression [29]. Therefore, we further investigated whether magnolin inhibits CXCL10‐induced senescence through modulation of the p38 signalling pathway. The results showed that treatment with magnolin effectively hindered CXCL10‐triggered phosphorylation of p38 (Figure 4l–n). These outcomes indicate that magnolin inhibited CXCL10‐induced cellular senescence and MMPs expression by suppressing p38 phosphorylation, ultimately leading to deceleration of CXCL10‐induced cellular senescence and ECM degradation.

### 3.4 Magnolin Mitigates Skin Ageing in Ex Vivo Human Skin

**FIGURE 5:**
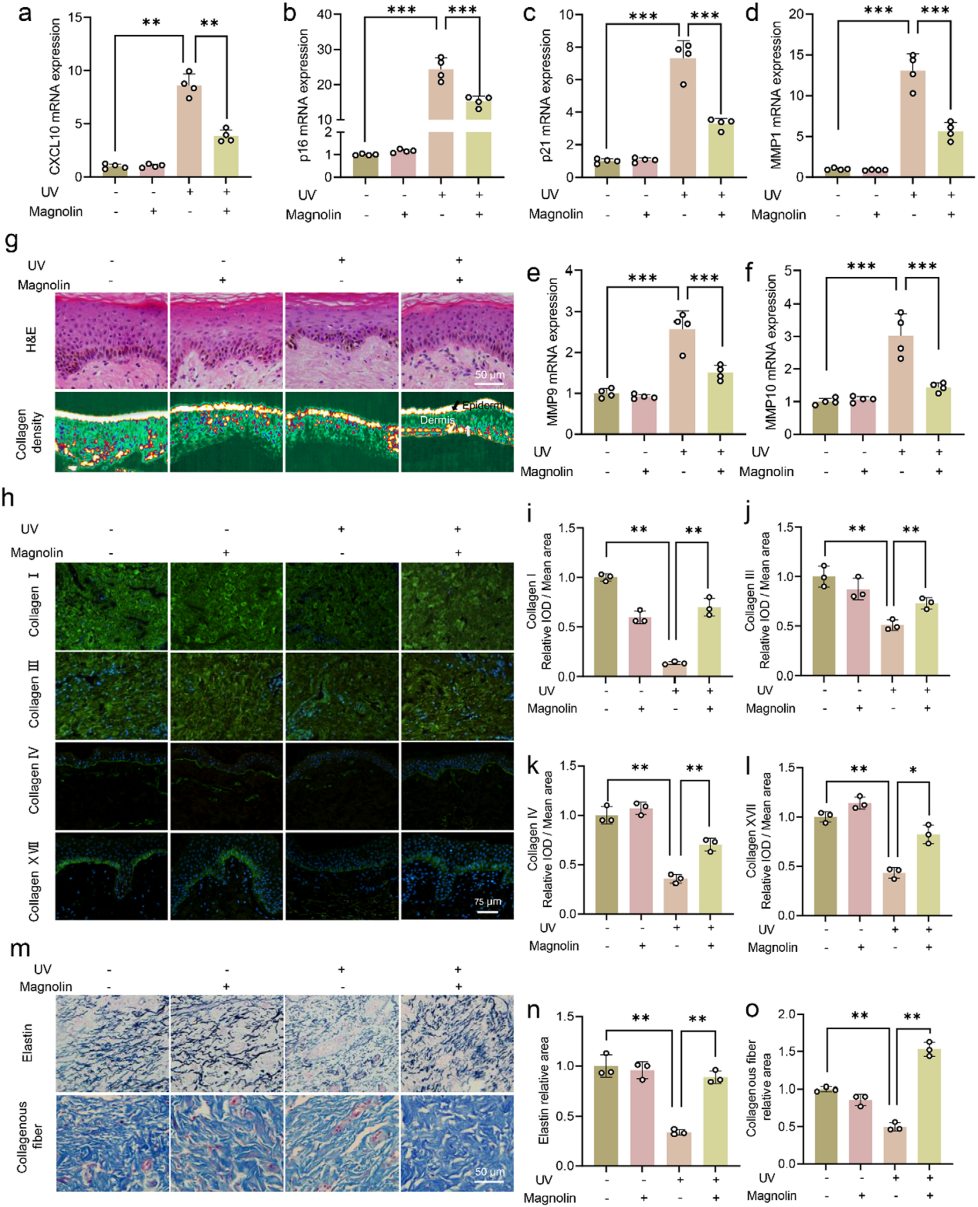
Magnolin suppresses skin ageing in ex vivo human skin. (a–f) QPCR detection of CXCL10, p16, p21, MMP1, MMP9 and MMP10 mRNA expression. (g) H&E staining and collagen density detection. (h) Immunofluorescence of Collagen I, Collagen III, Collagen IV and Collagen XVII. (i–l) The quantitative analysis of Figure 5h. (m) Victoria blue staining of elastic fibres and Masson staining of collagen fibres. (n, o) The quantitative analysis of Figure 5m. The isolated ex vivo human skin was cultured for 1 day before irradiation and magnolin administration. The irradiation doses were UVA (30 J/cm^2^) and UVB (50 mJ/cm^2^) for 3 consecutive days, and fresh culture medium containing 5 μg/mL magnolin was replaced after each irradiation. After treatment, total RNA was extracted for QPCR. Sections were embedded after fixation with 4% paraformaldehyde. (*n* = 3, *p < 0.05, **p < 0.01, ***p < 0.001).

Ultraviolet (UV) radiation is the most prevalent external factor that triggers skin ageing, and it has been reported that a significant upregulation of CXCL10 occurs in UV‐irradiated skin. Therefore, we conducted an investigation to assess the impact of magnolin on UV‐induced skin ageing using an ex vivo human skin model. As depicted in Figure 5, the mRNA expression of the chemokine CXCL10, matrix metalloproteinases (MMP1, MMP9 and MMP10), and ageing‐related proteins (p21 and p16) was significantly upregulated in UV‐treated ex vivo human skin. However, the addition of magnolin substantially suppressed the mRNA expression of these chemokines, metalloproteinases and ageing‐related proteins (Figure 5a–f).

Considering the inverse correlation between metalloproteinase expression and skin collagen and elastin content, we conducted H&E staining and various collagen detection assays on ex vivo skin samples subjected to different treatments. As shown in Figure 5g, UV exposure resulted in a significant reduction in epidermal thickness and collagen density. However, these effects were restored upon magnolin treatment. Given that MMP1 targets Collagen I and Collagen III, while MMP9 and MMP10 target Collagen IV and Collagen VII, respectively, we evaluated the expression levels of Collagen I, Collagen III, Collagen IV, Collagen XVII, collagen fibres and elastic fibres following UV exposure with or without magnolin intervention. The results demonstrated a substantial inhibition of the aforementioned protein expressions due to UV exposure. However, the magnolin intervention effectively restored their levels (Figure 5h–o), thereby further confirming its efficacy against UV‐induced skin ageing.

### 3.5 Magnolin Mitigates Facial Skin Ageing

**FIGURE 6:**
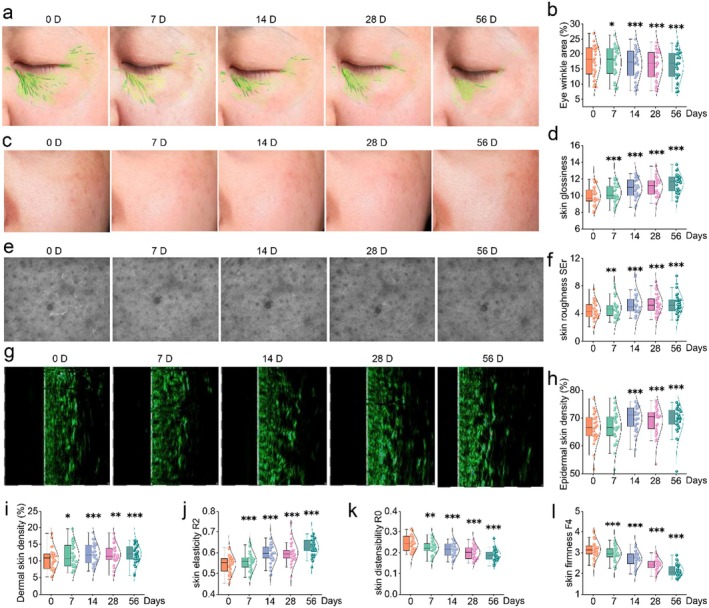
Magnolin inhibits facial skin ageing. (a) Wrinkles under the eyes. (b) The quantitative analysis of the eye wrinkle area. (c) Skin glossiness. (d) The quantitative analysis of skin glossiness value. (e) Roughness of cheek skin. (f) The quantitative analysis of the cheek skin roughness SEr value. (g) Skin epidermal layer density (UC22 imaging). (h) The quantitative analysis of skin epidermal layer density. (i) The quantitative analysis of skin dermal layer density. (j) Skin elasticity R2 value. (k) Skin distensibility value. (l) Skin firmness F4 value. (*n* = 30. *p < 0.05, **p < 0.01, ***p < 0.001).

To further investigate the anti‐ageing effect of magnolin on human skin, a total of 32 participants with sensitive skin were subjected to a 56‐day treatment with magnolin, ultimately collecting complete data from 30 participants. Clinical manifestations associated with skin ageing, including under‐eye wrinkles, skin glossiness, skin roughness, skin density, skin elasticity, distensibility and firmness, were assessed at 7, 14, 28 and 56 days. As depicted in Figure 6a,b, the application of magnolin for a duration of 56 days resulted in a significant reduction in the proportion of wrinkles under the eyes by 11.37%, an increase in overall skin glossiness by 15.26% (Figure 6c,d), as well as an enhancement in SEr value representing improved parameters related to skin roughness by 22.94% (Figure 6e,f). Moreover, there was a notable increase in skin density by 3.69% (Figure 6g–i), an elevated R2 value indicating enhanced elasticity by approximately 12.71% (Figure 6j), and an increased stretching height represented by R0 value which rose up to 16.89% (Figure 6k). Additionally, there was also a decrease of F4 value reflecting improved firmness reduced by 24.26% (Figure 6l) after using magnolin for the specified duration. Collectively, these results suggest that the application of magnolin possesses certain mitigating effects against human facial ageing.

## 4 Discussion

**FIGURE 7:**
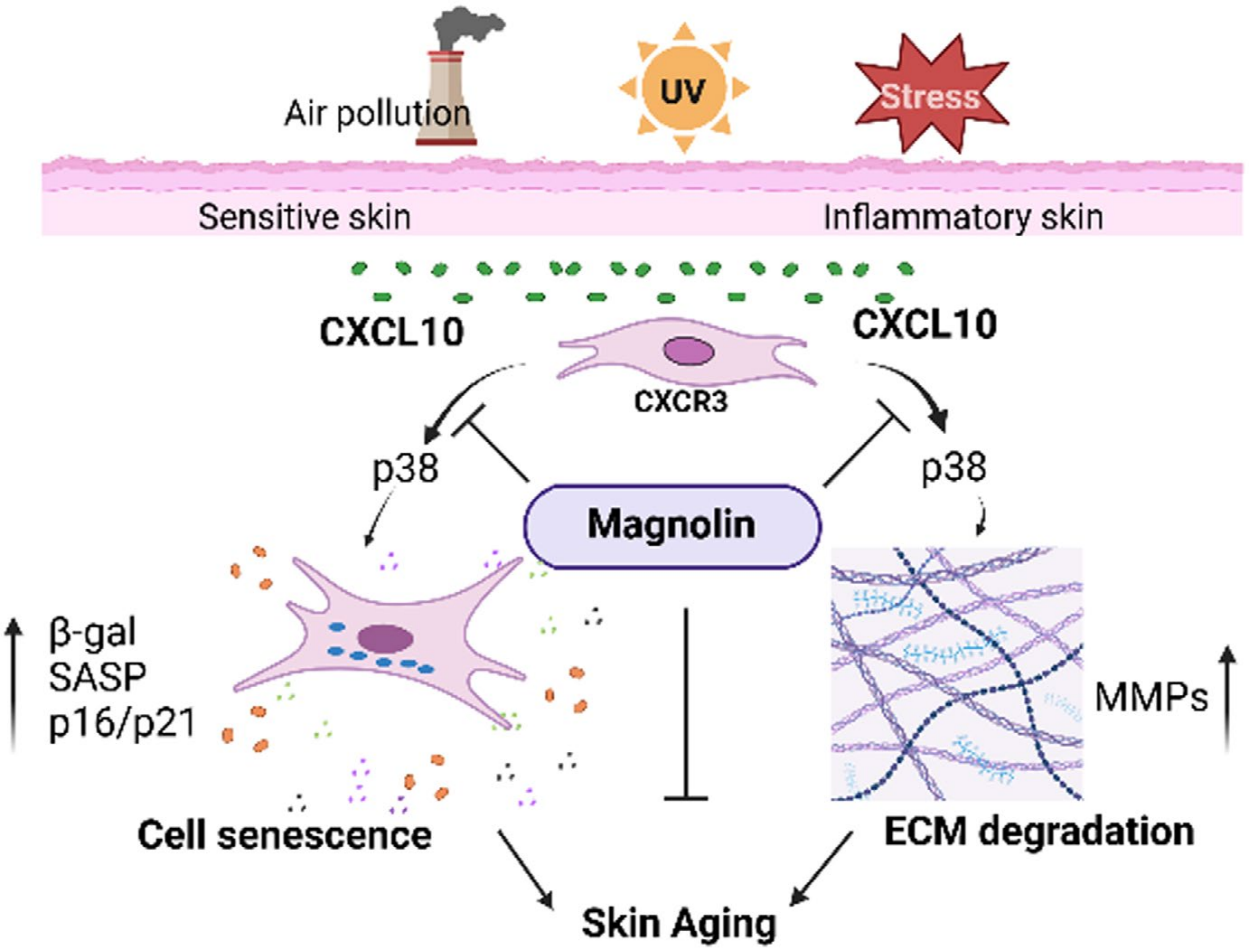
Magnolin mitigates CXCL10‐induced skin ageing. The skin can undergo chronic inflammation due to external factors such as exposure to sunlight, environmental pollution and psychological stress. During this process, CXCL10 plays a significant role in promoting skin fibroblast senescence and MMP‐induced ECM degradation through CXCR3, ultimately leading to skin ageing. Magnolin can mitigate this process through the inhibition of the CXCL10/p38 signalling pathway.

Inflammation is widely recognised as a significant contributor to skin ageing, necessitating the elucidation of its underlying mechanisms in order to prevent not only cutaneous ageing but also systemic ageing. Our experimental findings have identified CXCL10, a chemokine, as an inducer of skin ageing through the promotion of fibroblast cell senescence and MMPs expression. Subsequently, we conducted a screening of various plant extracts for their potential anti‐CXCL10‐induced skin ageing properties and discovered that magnolin from the *Magnolia biondii* flower extract effectively inhibits CXCL10‐induced β‐gal activity, as well as p21/p16 and MMP expression. This inhibition is mediated by the suppression of the p38 signalling pathway. Furthermore, we have validated the anti‐ageing efficacy of magnolin using ex vivo human skin exposed to UV radiation and human sensitive facial skin (Figure 7).

In this study, we screened 20 inflammatory factors induced by external factors in skin cells [30], which are common inflammatory factors expressed in inflammatory and sensitive skin [31]. The results highlighted that CXCL10 can increase the activity of β‐gal and the expression of ageing‐related genes, p21 and p16, which serve as key indicators of cell senescence [32]. Therefore, our subsequent studies focused on CXCL10 despite the potential of other inflammatory factors such as IL‐1α, IL‐1β and TNF‐α to induce MMPs expression.

Chemokines play a significant role in recruiting inflammatory cells and act as intermediaries and signal amplifiers in the process of inflammation [33, 34]. Recent studies have shown that chemokines, including CXCL9 [17] and CXCL5 [16], can induce cell senescence in ageing endothelial and mouse embryonic cells, respectively. These findings suggest that chemokines not only have chemotactic properties but also have the function of inducing cell senescence. Our study demonstrated that CXCL10 promotes skin ageing by increasing β‐gal activity, upregulating the expression of ageing‐related genes p21 and p16, and enhancing the expression of MMP1, MMP9 and MMP10. In this study, we conducted preliminary investigations to explore the potential of CXCL10 in inducing skin ageing via its receptor CXCR3 pathway. However, further studies are warranted to elucidate the underlying mechanisms.

Studies have shown that CXCL10 is highly expressed in various ageing‐related diseases, and its expression is induced by stimuli such as UVB, air pollution particles and TNF‐α, which are common in chronic inflammatory skin, particularly sensitive skin [11, 17, 35, 36, 37, 38, 39, 40]. Consistently, we also demonstrated that UV exposure can induce a significant increase in CXCL10 in ex vivo human skin, demonstrating the crucial role of CXCL10 in UV‐induced inflammaging. Inhibiting CXCL10‐induced skin ageing is thus critical for the treatment of sensitive skin, which not only resolves external stimuli‐induced inflammation but also mitigates chronic inflammatory‐mediated skin ageing. Therefore, 32 participants with sensitive skin were recruited to be treated with magnolin for 56 days, and the clinical manifestations of skin ageing such as wrinkles under the eyes, skin glossiness, skin roughness, skin density, skin elasticity, distensibility and skin firmness were significantly ameliorated, indicating the important role of CXCL10‐induced skin ageing in sensitive skin.

Magnolin is a naturally occurring and multi‐bioactive lignan molecule. It has been reported that magnolin exhibits anti‐inflammatory effects on chondrocytes via the NF‐κB pathway for attenuating anterior cruciate ligament transection‐induced osteoarthritis [41] and protects against contrast‐induced nephropathy in rats via antioxidation and antiapoptosis [42]. A recent study reported that magnolin from *Magnolia kobus* extract inhibited LPS‐induced pro‐inflammatory cytokines [43]. All these studies indicate the potential anti‐inflammaging function of magnolin. A published study has reported that the activation of CXCR3 induced p38 phosphorylation during the process of Japanese encephalitis virus infection [44]. Furthermore, another study demonstrated that CXCL10 induced cell migration through the CXCR3/p38‐MAPK pathway [45]. In our study, our screening of plant extracts identified magnolin from *Magnolia biondii* flower extract as a potent mitigant of CXCL10‐induced skin ageing. This mitigation is mediated by the suppression of the p38 signalling pathway. However, further investigation is warranted to fully elucidate the role of magnolin in combating skin ageing. Overall, this study highlights the crucial role of CXCL10 in inducing skin inflammaging and provides a new insight that magnolin can target and alleviate skin inflammaging.

## Author Contributions

**Cheng Wang:** investigation (equal), methodology (equal), visualization (equal), writing – original draft (equal), writing – review and editing (equal). **Xiaoyun Hu:** methodology (equal), project administration (equal), writing – original draft (equal), writing – review and editing (equal). **Tianlin Song:** investigation (equal), methodology (equal), writing – original draft (equal), writing – review and editing (equal). **Fan Hu:** writing – original draft (equal). **Le Du:** writing – original draft (equal). **Chenqiong Yan:** writing – original draft (equal). **Tianwei Shen:** writing – original draft (equal). **Nihong Li:** writing – original draft (equal). **Wei Yang:** writing – original draft (equal). **Li Li:** software (equal), validation (equal), visualization (equal). **Nian Deng:** software (equal), validation (equal), visualization (equal). **Xingwu Jiang:** project administration (equal), writing – original draft (equal), writing – review and editing (equal). **Yelin Wu:** funding acquisition (equal), project administration (equal), visualization (equal), writing – original draft (equal), writing – review and editing (equal). **Rui Ye:** project administration (equal), resources (equal), software (equal), visualization (equal), writing – original draft (equal), writing – review and editing (equal).

## Conflicts of Interest

The authors declare no conflicts of interest.

## Supporting information


Appendix S1.

## Data Availability

The main data supporting the results in this study are available within the paper and its Supporting Information Data available on request from the authors. The data that support the findings of this study are available from the corresponding author upon reasonable request.
