# Supplementary material for: Magnolin Mitigates Skin Ageing Through the CXCL10/p38 Signalling Pathway
Source: J Cell Mol Med. 2025 May 19;29(10):e70507. doi: 10.1111/jcmm.70507 (PMC12086986; doi:10.1111/jcmm.70507)
Supplement: Supplementary file 1 — Appendix S1. [file JCMM-29-e70507-s001.docx]

Magnolin mitigates skin aging through the CXCL10/p38 signaling pathway

Cheng Wang ^1,†^, Xiaoyun Hu ^2,4,†^, Tianlin Song ^1,†^, Fan Hu ^2,4^, Le Du ^2^, Chenqiong Yan ^2,4^, Tianwei Shen ^2,4^, Nihong Li ^2^, Wei Yang ^1^, Li Li ^5^, Nian Deng ^1^, Xingwu Jiang ^3,*^, Yelin Wu ^1,*^ and Rui Ye ^2,4,5,*^

^1^ Shanghai Tenth People's Hospital, Tongji University School of Medicine, Shanghai 200072, P. R. China

^2^ UNISKIN Research Institute on Skin Aging, Inertia Shanghai Biotechnology Co., Ltd., Shanghai 200021, P. R. China

^3^ Department of Materials Science and State Key Laboratory of Molecular Engineering of Polymers, Fudan University, Shanghai 200433, P. R. China

^4^ DermaHealth Shanghai Biotechnology Co., Ltd., Shanghai 200021, P. R. China

^5^ Department of Dermatology & Venerology, Center of Cosmetic Safety and Efficacy Evaluation and NMPA Key Laboratory for Human Evaluation and Big Data of Cosmetics, West China Hospital, Sichuan University, Chengdu 610041, P. R. China

* Correspondence: sk_wuyelin@tongji.edu.cn (Y.W.); rye@inertiabiotech.com (R.Y.); xwjiang@fudan.edu.cn (X.J.)

^†^ These authors contributed equally to this work.

**Table S1.** Oligonucleotides of primers

| **Name of Gene** | **Primers** |
| --- | --- |
| Human GAPDH | F: CTTAGCACCCCTGGCCAAG  R: TGGTCATGAGTCCTTCCACG |
| Human MMP1 | F: AAAATTACACGCCAGATTTGCC  R: GGTGTGACATTACTCCAGAGTTG |
| Human MMP3 | F: CTGGACTCCGACACTCTGGA  R: CAGGAAAGGTTCTGAAGTGACC |
| Human MMP9 | F: TGTACCGCTATGGTTACACTCG  R: GGCAGGGACAGTTGCTTCT |
| Human MMP10 | F: TGCTCTGCCTATCCTCTGAGT  R: TCACATCCTTTTCGAGGTTGTAG |
| Human p16 | F: GATCCAGGTGGGTAGAAGGTC  R: CCCCTGCAAACTTCGTCCT |
| Human p21 | F: TGTCCGTCAGAACCCATGC  R: AAAGTCGAAGTTCCATCGCTC |
| CXCL10 | F: GTGGCATTCAAGGAGTACCTC  R: TGATGGCCTTCGATTCTGGATT |


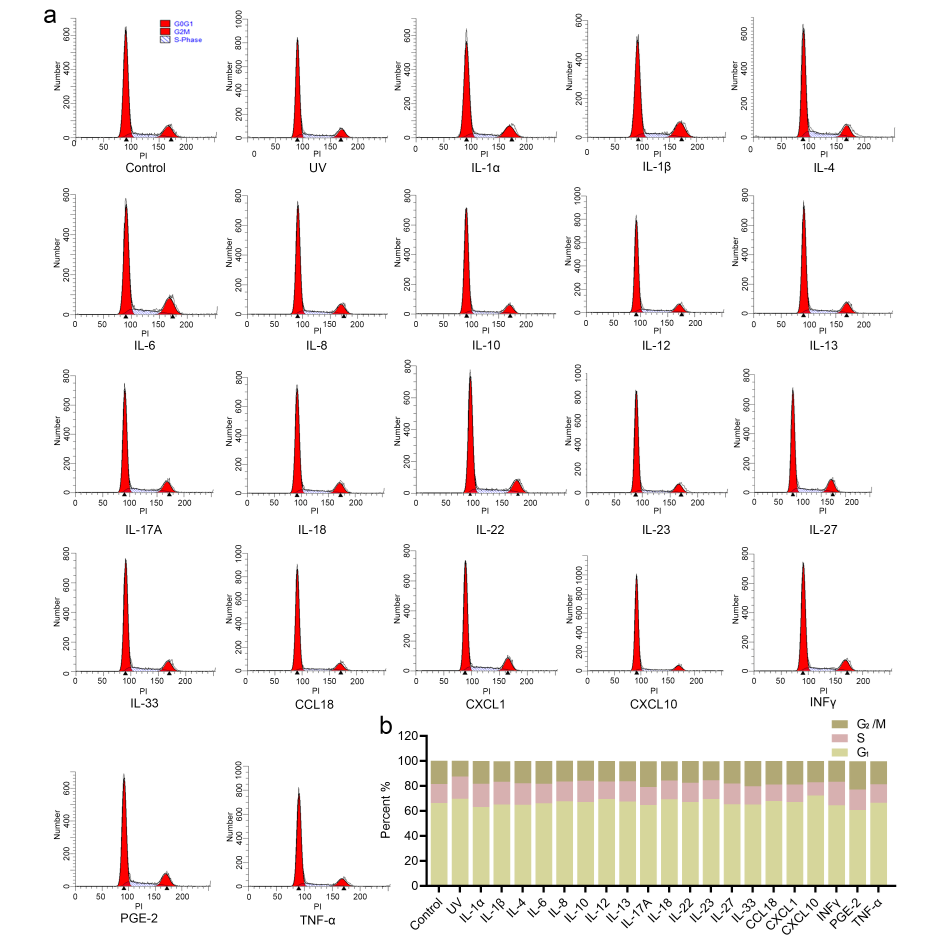


**Figure S1.** **Inflammatory factors induce cell cycle arrest**. (**a**) HSF was incubated with 100 ng/mL inflammatory factors for 24 h, and then stained with PI and detected by flow cytometry. (The positive control was irradiated with UVB at 60 mJ/cm^2^). (**b**) The statistics analysis of cytometry in A. (n=3)


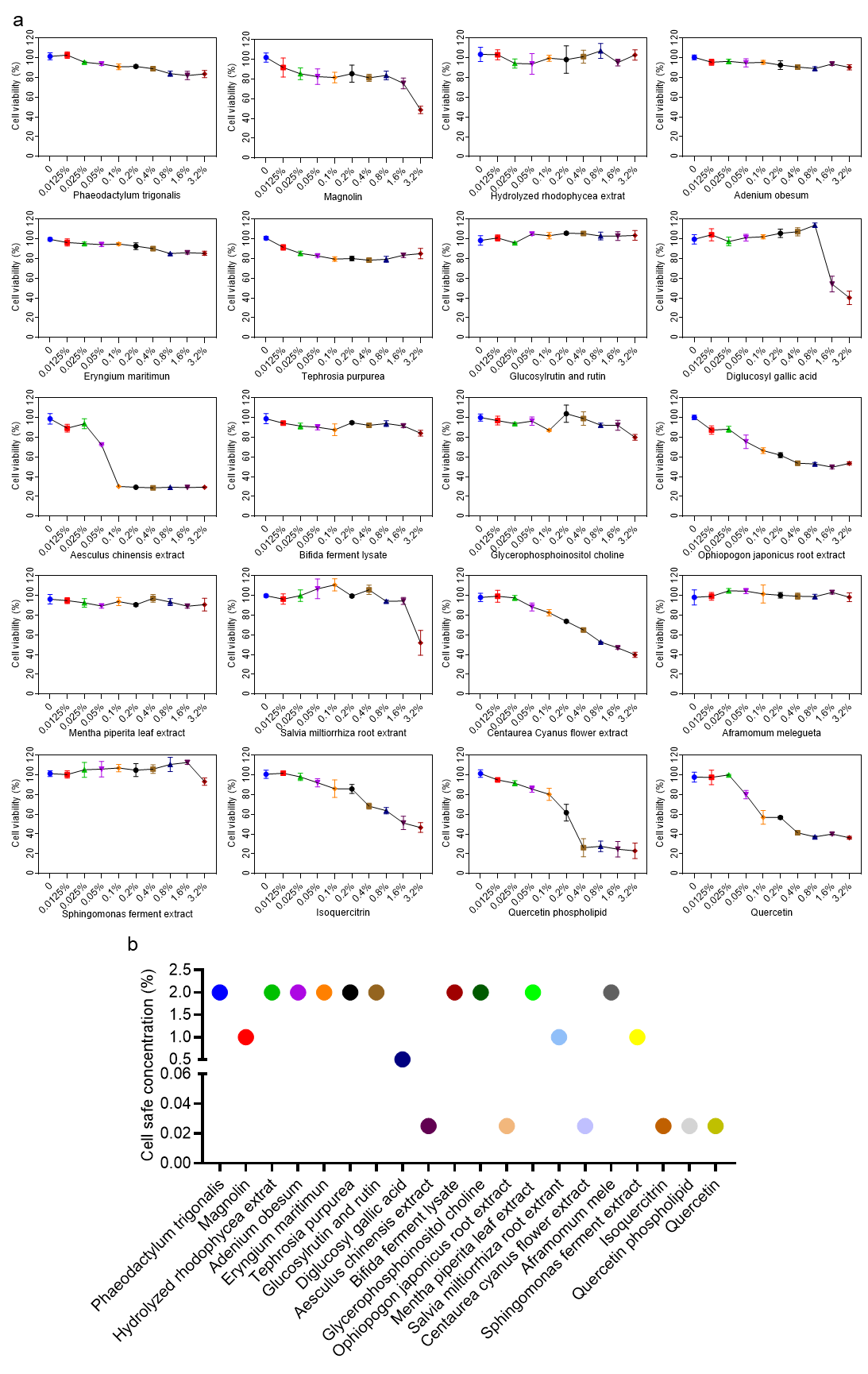


**Figure S2. Evaluation of the biologically safe concentration of active ingredients.** (**a**) HSF cells were seeded in a 96-well plate for 24 h and treated with different concentrations of active ingredients to determine their biologically safe concentration. (**b**) Safe concentration of each active ingredients of Figure S2a (n=6).


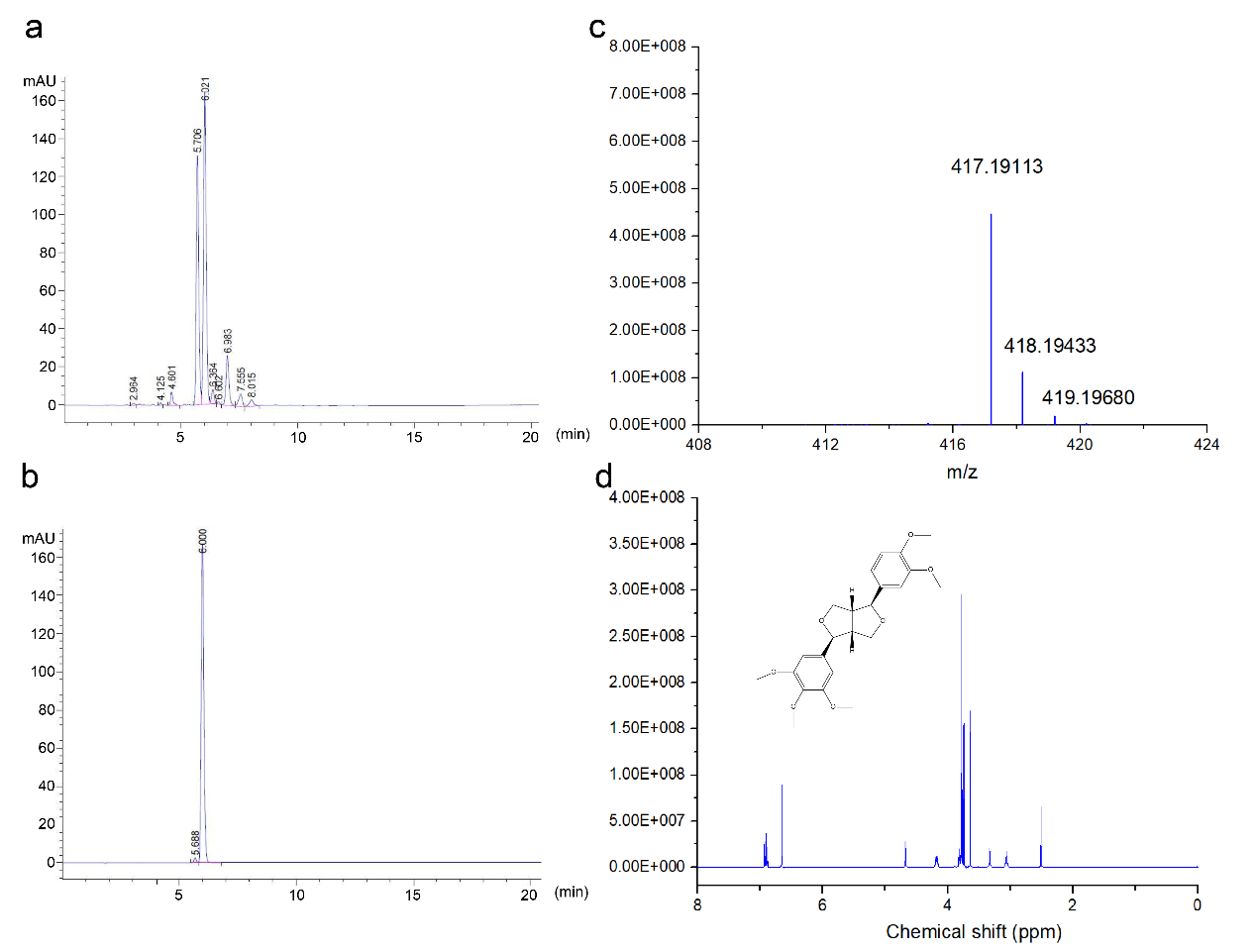


**Figure S3. Identification of active ingredients.** (**a**): HPLC of Magnolia biondii flower extract. (**b**): HPLC of Magnolin. (**c**): HR-MS of Magnolin. (**d**):^1^H NMR of Magnolin.

**
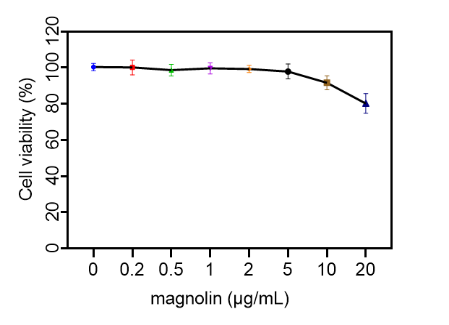
**

**Figure S4. Evaluation of the biologically safe concentration of magnolin.**


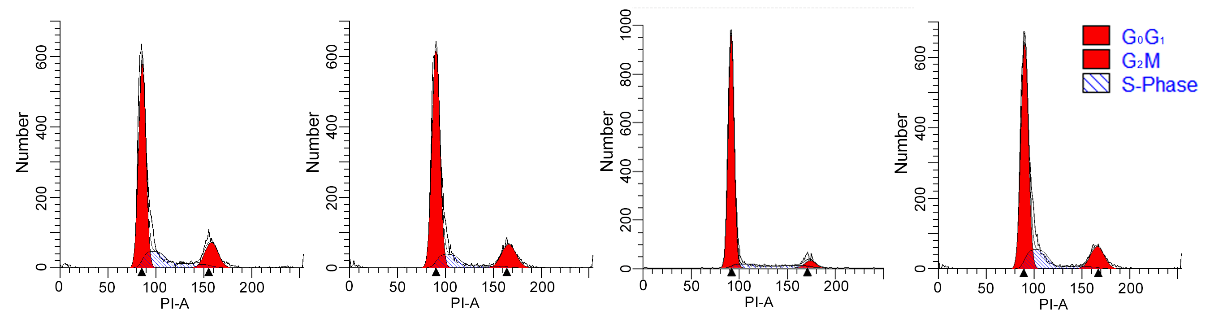


**Figure S5. Magnolin inhibits CXCL10-induced cell cycle arrest.** HSF was incubated with 200 ng/mL CXCL10 and 5 μg/mL magnolin for 24 h, and then stained with PI and detected by flow cytometry.
